# Student Perspectives on Marketing the Podiatry Profession and Course Promotion: A Mixed Methods Study

**DOI:** 10.1002/jfa2.70063

**Published:** 2025-07-21

**Authors:** Michelle R. Kaminski, Caroline Robinson, Glen A. Whittaker, Malia Ho, Daniel R. Bonanno, Shannon E. Munteanu, Mollie Dollinger, Sia Kazantzis, Xia Li, Ryan S. Causby, Mike Frecklington, Steven Walmsley, Vivienne Chuter, Sarah L. Casey, Matthew Cotchett

**Affiliations:** ^1^ Discipline of Podiatry School of Allied Health, Human Services and Sport La Trobe University Melbourne, Victoria Australia; ^2^ Department of Podiatry Monash Health Melbourne, Victoria Australia; ^3^ School of Primary and Allied Health Care Monash University Melbourne, Victoria Australia; ^4^ School of Allied Health, Exercise and Sports Sciences Charles Sturt University Albury, New South Wales Australia; ^5^ Faculty of Health Sciences Curtin University Bentley, Western Australia Australia; ^6^ Melbourne School of Health Sciences Department of Physiotherapy The University of Melbourne Melbourne, Victoria Australia; ^7^ Mathematics and Statistics School of Computing, Engineering and Mathematical Science La Trobe University Melbourne, Victoria Australia; ^8^ Allied Health and Human Performance Unit University of South Australia Adelaide, South Australia Australia; ^9^ Department of Podiatry School of Clinical Sciences Faculty of Health and Environmental Sciences Auckland University of Technology Auckland New Zealand; ^10^ Discipline of Podiatry School of Clinical Sciences Faculty of Health Queensland University of Technology Brisbane, Queensland Australia; ^11^ School of Health Sciences Western Sydney University Campbelltown, New South Wales Australia; ^12^ Discipline of Podiatry School of Health Sciences Western Sydney University Campbelltown, New South Wales Australia

**Keywords:** career choice, health workforce, marketing, podiatry, students

## Abstract

**Background:**

The decline in podiatry student enrolments at universities across Australia and New Zealand presents a workforce crisis that threatens the profession's sustainability and the delivery of essential healthcare services to communities. Recent data highlight a limited understanding of the podiatry profession among allied health students. There is now a clear need for strategies to address negative stereotypes and to build knowledge of the profession's scope of practice, career opportunities, job prospects and earning potential. As part of a larger research initiative, this study explored student perspectives on marketing the podiatry profession to increase student enrolments.

**Methods:**

A convergent mixed methods study design was employed. Participants included students enrolled in (i) podiatry and (ii) relevant ‘non‐podiatry’ health, sport or science programs at nine Australian universities and one New Zealand university. Data were collected via an online survey (278 podiatry students and 553 non‐podiatry students responding) and two online workshops with nine first‐year podiatry students. Quantitative data were analysed using descriptive statistics and regression models, while qualitative data underwent inductive thematic analysis by three independent assessors. We integrated the data by exploring the connections between the quantitative and qualitative findings.

**Results:**

Findings revealed that over 40% of podiatry students had initial concerns about their course and 34.2% had considered leaving. Instagram and Facebook were identified as the most influential social media platforms for sourcing information about courses and careers. Four over‐arching themes emerged as important marketing strategies for increasing student enrolments: (i) enhance the visibility, perception and advocacy of podiatry; (ii) emphasise holistic and diverse practice in podiatry; (iii) enable early exposure and experience of podiatry practice and (iv) improve course entry pathways and flexibility.

**Conclusions:**

An evidence‐based approach is required to enhance the visibility and appeal of podiatry as a career. Strategies should focus on addressing misconceptions about the discipline, expanding promotional efforts to broader audiences, leveraging relevant media platforms, reducing financial and academic barriers for prospective students, and improving study flexibility. Strengthening enrolments and reducing attrition are fundamental to ensuring the sustainability and growth of the podiatry profession in Australia and New Zealand.

## 1 Background

The podiatry profession in Australia and New Zealand, along with broader global trends [1, 2, 3], is facing a critical workforce challenge, primarily driven by a decline in student enrolments in undergraduate programs [4, 5, 6]. Despite the well‐established value of podiatry in healthcare, the apparent diminishing interest or lack of awareness about the profession among prospective students [1, 2, 4] threatens the viability of university programs. This downward trend poses significant concerns for the sustainability and future growth of the profession [1, 2, 4]. A reduced inflow of podiatry graduates to the profession will risk workforce shortages, increase burnout [7] and compromise healthcare delivery [4]. The consequences of these shortages are particularly pronounced in rural and remote areas, where gaps in podiatry services exacerbate health inequities and limit access to essential care [8, 9, 10].

Our recent study [4] found that allied health students have a limited understanding of the podiatry profession and highlighted actions to address negative podiatry stereotypes, shift perceptions of podiatry and build knowledge of the profession's scope of practice, career pathways/opportunities, job prospects and earning potential [1, 2, 4]. These insights provide a foundation for promoting podiatry programs; however, a deeper exploration of students' perspectives on marketing the profession is needed. Understanding student viewpoints could provide a nuanced perspective on which messages resonate and which may deter prospective students, enabling more effective marketing planning. Such research is essential for developing evidence‐based marketing strategies and campaigns that enable key stakeholders to effectively showcase the aspects of podiatry which prospective students will find appealing. Through strategic marketing, we aim to address the student enrolment crisis that threatens the viability of podiatry programs, a key factor contributing to the shortage of podiatry professionals [1, 2, 4]. By attracting students whose career aspirations align with the rewards of the profession, this could mitigate workforce shortages by fostering a continuous stream of future podiatrists. These initiatives are likely to have a broader impact on improving the sustainability and growth of the podiatry profession. This study serves as an extension of our previous work [4], with a distinct emphasis on marketing the podiatry profession from the student perspective. Therefore, this study aimed to explore student perspectives on marketing the podiatry profession to inform strategies for increasing student enrolments and sustaining the workforce in Australia and New Zealand.

## 2 Methods

### 2.1 Ethics Approval

This study was approved by the relevant institutional Human Research and Ethics Committees (HEC21057; 21/161; 22978; H21077; 2021/043; H‐2021‐0276; 203889; 2021/ET000372; H14404), and all participants provided informed consent prior to data collection [4].

### 2.2 Study Design and Reporting

Employing a convergent mixed methods design, this study gathered and analysed both quantitative and qualitative data concurrently, with integration achieved through merging [11, 12]. The findings are presented in accordance with the consolidated criteria for reporting qualitative research (COREQ) framework and the Good Reporting of A Mixed Methods Study (GRAMMS) checklist [13, 14]. The completed GRAMMS checklist is available in Supporting Information S1.

### 2.3 Research Team

Our research team was comprised of academics with diverse expertise in quantitative and qualitative research in foot and ankle pathology, chronic disease, health promotion and culturally responsive practice [4]. This diversity ensured robust analysis and interpretation of findings.

### 2.4 Participants

#### 2.4.1 Online Survey

Students from first to third (or fourth) year in podiatry and relevant non‐podiatry programs at nine Australian universities and one New Zealand university were invited to participate in an online survey. A ‘relevant non‐podiatry program’ encompassed any health, sport or health‐related science program from universities that offered a podiatry program (students within these programs hereinafter referred to as ‘non‐podiatry students’). The non‐podiatry programs included nursing, physiotherapy, exercise science, occupational therapy, speech pathology, dietetics/nutrition, orthoptics, prosthetics and orthotics and various science programs (e.g., health science and biomedicine).

#### 2.4.2 Workshops

Invitations to participate in the online workshops were extended to all first‐year podiatry students from four participating universities [4].

### 2.5 Sample Size

An a priori sample size calculation determined that 264 participants were required for the podiatry student survey portion of this study. This calculation was based on the following: (i) 880 podiatry students across all universities, (ii) expected response rate of ∼30%, (iii) a confidence level of 95% and (iv) a margin of error of 5% [4, 15]. Our sample size for the workshops (*n* = 9) aligned with qualitative research guidelines [16, 17] to ensure that diverse student perspectives were captured, while fostering focused interactions.

### 2.6 Recruitment

Podiatry Program Leads from 10 university podiatry programs across Australia and New Zealand were approached via email with an invitation to participate in this study. Only one institution chose not to participate. Following confirmation of participating podiatry programs, invitations were also extended to Program Leads from related non‐podiatry disciplines at those universities.

### 2.7 Data Collection

#### 2.7.1 Online Survey

Online survey data were collected and managed using REDCap electronic data capture tools hosted at La Trobe University [18, 19]. REDCap (Research Electronic Data Capture, Vanderbilt University, USA) is a secure web‐based software platform designed to support data capture for research studies [18, 19]. The online survey consisted of two separate versions: one for podiatry students and one for non‐podiatry students. Both surveys included a mix of open and closed questions and are available as supplementary files in a previous publication [4]. The surveys included questions on demographics as well as the motivators and barriers for the students' respective career choices. The podiatry survey also included questions on marketing of the profession, whereas the non‐podiatry survey included questions on knowledge and perceptions of the podiatry profession. The podiatry survey component was initially piloted on first‐year podiatry students (*n* = 30; response rate of 29.7%) at three Australian universities. Based on feedback from the pilot, the survey was refined to create the version used in this study.

An email invitation containing a survey link was sent to podiatry and non‐podiatry students by a university staff member, such as the year‐level coordinator. Interested students were invited to complete the survey at their own convenience. To maximise participation, reminder emails (three in total) were sent weekly over a 4‐week data collection period. The survey was accessible online from 7 March 2022 to 14 April 2022.

#### 2.7.2 Workshops

The workshops were conducted via Zoom videoconferencing [20] and followed a co‐design workshop approach known as ‘CoLabs’ [21]. Scaffolded participatory design activities were employed in the workshops, incorporating a range of activities designed to foster student reflection and feedback, as well as generate ideas [21]. Our previous publication provides full details of the workshop activities [4]; however, a brief overview is provided here for context. In the first activity, students used Jamboard electronic whiteboard (Google Inc.) to create a mind map capturing their initial thoughts and associations with the word ‘podiatry’. The second activity required students to contribute to an online discussion forum using the Padlet software, where students reflected on personal motivators and barriers related to studying podiatry and offered suggestions for improving how the profession is marketed. In the final activity, students collaborated in small groups to co‐create a recruitment campaign for podiatry. This included identifying key selling points, outlining dissemination strategies and highlighting unique aspects of the profession. All data generated from the workshop activities were collated in NVivo (Lumivero). All workshops were conducted within an 8‐day period, commencing in March 2022.

### 2.8 Data Analysis

#### 2.8.1 Quantitative Data

Descriptive statistics summarised participant characteristics and data obtained from the closed survey questions. Categorical data were presented as frequencies and percentages. Continuous data were expressed as mean (standard deviation, SD) or median (interquartile range, IQR). Parametric statistical methods were applied to normally distributed data, whereas non‐parametric methods were used for data that did not follow a normal distribution. To explore between‐group differences (e.g., podiatry and non‐podiatry students), independent samples *t*‐tests, Mann–Whitney *U* tests and/or Chi‐squared tests were used. Associations between personal and professional factors that influence perceptions on marketing strategies for podiatry and non‐podiatry students were explored using linear and/or logistic regression (depending on data type) and were expressed as odds ratios (ORs) and 95% confidence intervals (CIs). For missing data, casewise deletion was used for any variables included in regression models with missing data. All statistical analyses were conducted using R (version 4.3.2, R Foundation for Statistical Computing, Vienna, Austria). Statistical significance was set at the two‐sided conventional level of ≤ 0.05.

#### 2.8.2 Qualitative Data

Themes were developed from open questions in the online survey provided by podiatry (*n* = 278) and non‐podiatry (*n* = 553) students as well as qualitative data collected in the podiatry student workshops (*n* = 9). All data were initially collated in Microsoft Excel (Redmond, Washington, USA) and analysed using inductive thematic analysis by three independent assessors (CR, MH and MC). NVivo software (Lumivero) was used for coding, and final themes were established through consensus. Thematic analysis followed the six‐phase process described by Braun and Clarke [16]: (i) data familiarisation, (ii) initial coding, (iii) theme searching, (iv) reviewing themes, (v) defining and naming themes and (vi) manuscript preparation.

#### 2.8.3 Data Integration

Employing a convergent mixed methods design, this study gathered and analysed both quantitative and qualitative data concurrently, with integration achieved through merging [11, 12]. Specifically, we evaluated whether the results from each type of analysis were consistent or divergent. Furthermore, we highlighted which elements of the quantitative data were not addressed in the qualitative study and vice versa. Integration of findings derived from each analysis was undertaken by researchers MRK, CR, MC and MH. Tables that aligned findings from both datasets were used to enable comparisons and data integration.

## 3 Results

A total of 278 podiatry students and 553 non‐podiatry students participated in the online survey, with strong representation from all year levels (mean age 24.9 [SD, 8.5], 65.1% female for podiatry students and mean age 24.8 [SD, 8.2], 75.4% female for non‐podiatry students). Nine first‐year podiatry students from Australia (two females and two males) and New Zealand (five females) participated in the online workshops. Participant characteristics are provided in Table 1 and our previous publication [4]. The non‐podiatry group included students from 13 disciplines, with a notable proportion from physiotherapy (32.2%) and occupational therapy (29.1%). Overall, the majority of students were enrolled full‐time (92.4% of podiatry students and 85.9% of non‐podiatry students) and many held prior qualifications (45.7% of podiatry students and 39.4% of non‐podiatry students).

**TABLE 1:** Participant characteristics.

Variables	Podiatry (*n* = 278)	Non‐podiatry (*n* = 553)
Age, years, *mean* (SD)^a^	24.9 (8.5)	24.8 (8.2)
Sex, female, *n* (%)	181 (65.1)	417 (75.4)
Carer responsibilities, *n* (%)	37 (13.3)	91 (16.5)
Prior educational qualifications, *n* (%)^a^	127 (45.7)	218 (39.4)
Year of study, *n* (%)^a^		
First	76 (27.3)	152 (27.5)
Second	68 (24.5)	152 (27.5)
Third	81 (29.1)	152 (27.5)
Fourth	53 (19.1)	96 (17.4)
International student, *n* (%)^a^	17 (6.1)	19 (3.4)
Encountered barriers to studying podiatry, *n* (%)^a^ ^,^ ^b^	71 (25.5)	19 (3.4)
Concerns about studying podiatry, *n* (%)^a^	112 (40.3)	—
Considered leaving their course, *n* (%)^a^	95 (34.2)	147 (26.6)
Family commitments	19 (6.8)	35 (6.3)
Work commitments	10 (3.6)	35 (6.3)
Financial hardship	18 (6.5)	48 (8.7)
Health or stress	48 (17.3)	79 (14.3)
Study/life balance	43 (15.5)	70 (12.7)
Difficulties relating to workload	35 (12.6)	55 (9.9)
Personal reasons	20 (7.2)	37 (6.7)
Not enjoying the course	26 (9.4)	33 (6.0)
Course was not as expected	21 (7.6)	26 (4.7)
Change in mind regarding career path	29 (10.4)	45 (8.1)
Other	15 (5.4)	17 (3.1)
Social media platforms used the most, *n* (%)		
Instagram	204 (73.4)	364 (65.8)
Facebook	166 (59.7)	330 (59.7)
Twitter	21 (7.6)	27 (4.9)
TikTok	97 (34.9)	171 (30.9)
Snapchat	98 (35.3)	193 (34.9)
LinkedIn	31 (11.2)	47 (8.5)
Other	11 (4.0)	24 (4.3)

*Note:* Data are *n* (%), unless otherwise specified.

^a^ Missing data were for ‘Age’ (podiatry, *n* = 1), ‘Prior educational qualifications’ (podiatry, *n* = 1), ‘Year of study’ (non‐podiatry, *n* = 1), ‘International student’ (non‐podiatry, *n* = 1), ‘Encountered barriers to studying podiatry’ (podiatry, *n* = 25), ‘Concerns about studying podiatry’ (podiatry, *n* = 25) and ‘Considering leaving their course’ (podiatry, *n* = 25 and non‐podiatry, *n* = 80).

^b^ Non‐podiatry group only responded if reported that they had considered studying podiatry (*n* = 50).

**FIGURE 1:**
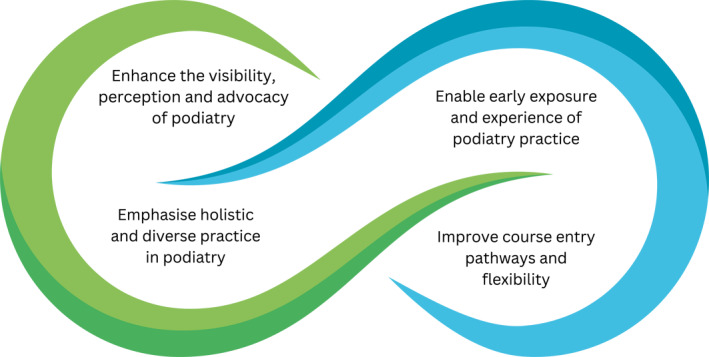
Overview of student perspectives on marketing the podiatry profession and course promotion.

The themes presented in this paper represent contributions from podiatry and non‐podiatry students, with a primary focus on student perspectives for marketing the podiatry profession and course promotion. The inter‐relationships between the themes are illustrated in Figure 1. Four over‐arching themes and 11 subthemes were identified. The over‐arching themes were as follows: (i) enhance the visibility, perception and advocacy of podiatry; (ii) emphasise holistic and diverse practice in podiatry; (iii) enable early exposure and experience of podiatry practice and (iv) improve course entry pathways and flexibility.

### 3.1 Theme 1. Enhance the Visibility, Perception and Advocacy of Podiatry

#### 3.1.1 Showcase the Diversity of Practice, Career Pathways/Opportunities, Job Prospects and Earning Potential via Social Media

Students recognised the potential of promoting the diversity of practice and career opportunities that are available within podiatry. Many students believed that other allied health professions, such as physiotherapy and occupational therapy, have been more effective in their marketing efforts, which negatively impacts prospective students' perceptions of podiatry. As one student explained:

Promote all aspects of podiatry … just think feet and it doesn't appeal to me but if you promoted it in a broader context and put it out there a lot more than it is at the moment, it may attract more students. I feel occupational therapy, physio[therapy] and speech [pathology] get a lot of coverage, but podiatry is something you [don’t] hear a lot about unless you are already interested in the field

(participant #266, female (F), second‐year podiatry student)

Quantitative findings supported these perspectives, highlighting the factors podiatry students found particularly attractive about the profession. Compared to the non‐podiatry students, podiatry students were significantly *more likely* to think that job prospects after graduation (OR 1.43, 95% CI 1.01 to 2.05, *p* = 0.050), the prospect of owning a business (OR 2.15, 95% CI 1.73 to 2.69, *p* < 0.001) and the ability to work in hospitals (OR 1.48, 95% CI 1.18 to 1.87, *p* = 0.001) were attractive factors.

Podiatry students believed that emphasising the growth of podiatry's scope of practice, the flexibility and security of employment and diverse work settings would appeal to prospective students more effectively and encourage a greater interest in podiatry:

… mention the growth in the field and the abundance of career opportunities and progression that can be made. That would stand out to many prospective students

(participant #169, F, first‐year podiatry student)

[I] didn’t originally know much about the job prospects as well as not knowing in depth what the role of a podiatrist entails … the profession stretches across multiple disciplines (i.e., sports, paediatrics, podiatric surgery etc)

(participant #104, F, second‐year podiatry student)

Students perceived that podiatry is not well promoted as a career choice as reflected in both the quantitative and qualitative analysis. When asked about the promotion of podiatry courses and career opportunities compared to other allied health fields, both podiatry students (33.1 out of 100 for courses and 42.2 out of 100 for career opportunities) and non‐podiatry students (25.9 out of 100 for courses and 26.0 out of 100 for career opportunities) identified a deficit of attention:

Appears to be a smaller profession with less exposure/promotion in comparison to other health professions

(participant #383, F, first‐year physiotherapy student)

Insufficient knowledge about the podiatry discipline, including diversity of practice, career pathways/opportunities, job prospects, earning potential and education pathways, was evident amongst students:

To be honest, despite having been to a podiatrist numerous times in my life, and needing to wear orthotics … I don't have a clue about career options, or what or how long the training would take

(participant #515, F, second‐year occupational therapy student)

I did not know anything about podiatry and wasn't that interested until my sister told me about what conditions they come across and how they help people. Everything to stress factors and how gait or even pain on your foot can have big effects on the rest of the body

(workshop #2, first‐year podiatry student)

Many students reported that there is a need to broaden the public's understanding of the role of podiatry within the healthcare system. As one student noted:

Showing exactly where we fit within healthcare as a profession and showing the full extent of what podiatrists can do. Changing the old perception that it is just toenails, corns and callus

(participant #181, male (M), third‐year podiatry student)

The students perceived that to effectively educate the public about the podiatry profession and promote podiatry programs and career opportunities to prospective students, it is crucial to utilise media channels that offer the greatest reach to the target audience. Social media platforms, in particular, were believed to be well‐suited for disseminating information about the podiatry profession, including scope of practice and career opportunities, and are also a valuable forum to address negative stereotypes and misconceptions:

Promotion on social media would be a great step forward to help attract more interest in the program as many people do not even know what a podiatrist is/what being a podiatrist involves

(participant #104, F, second‐year podiatry student)

Students also highlighted the expansive reach of social media, emphasising its potential to engage prospective students to consider podiatry:

Social media is massive—this needs to be used to reach younger and in some instances, mature‐age students

(participant #476, F, fourth‐year podiatry student)

#### 3.1.2 Support Podiatrists to Promote the Profession

Students identified that practising podiatrists are well‐positioned to promote podiatry as a potential career choice for a prospective student of any age. They likely possess the knowledge required to be effective ambassadors for the profession, offering valuable first‐hand insights into the diverse opportunities within podiatry practice. For many, enroling in a healthcare program of study represents the start of a lifelong journey, and a podiatrist may be the person to inspire a prospective student. One student shared how their interaction with a podiatrist shaped their decision to pursue this career after an initial setback:

I did not get into medicine, and I believed this course [podiatry] would give me a very similar, if not the same experience, as being a medical student … [I] wanted to work with similar cases, as I’ve been treated on [by a podiatrist] my whole life

(workshop #1, first‐year podiatry student)

Students also highlighted the opportunity for podiatry practitioners to passively promote the profession by placing pamphlets and posters in patient waiting areas. Careers advice and podiatry course information could also be shared in this way, with minimal impact on a busy practitioner's time:

More education on what podiatrists do; there isn't really easy access to finding out information. Maybe some posters on the wall with the interesting factors or some talks you could attend if you were interested in finding out more

(workshop #2, first‐year podiatry student)

Based on our quantitative data, many of the non‐podiatry students elected to study their chosen course because they had a first‐hand positive experience with the profession (e.g., they may have been treated by a physiotherapist previously and recovered well). In contrast, podiatry students were *more likely* to choose podiatry if they failed to get into another course (OR 1.59, 95% CI 1.34 to 1.90, *p* < 0.001) or they heard about the profession from a current podiatry student (OR 1.24, 95% CI 1.03 to 1.49, *p* = 0.026) compared to students in other health disciplines.

#### 3.1.3 Focus on Patient Experiences

Patient experiences may play a crucial role in the promotion of the podiatry profession. Positive interactions between patients and podiatrists can significantly influence public perception and awareness of the field. As highlighted by a podiatry student:

Patients with positive experiences with a professional are likely to recommend the profession to family and friends. For me, word of mouth is the best way to pique people's interest in certain careers

(participant #604, F, second‐year podiatry student)

When patients share their positive experiences with others, they become effective ambassadors for the profession, helping to generate interest among potential future podiatrists. Students thought that word of mouth, fuelled by these first‐hand accounts, may be a powerful tool for reaching new audiences and encouraging them to consider a career in podiatry.

### 3.2 Theme 2. Emphasise Holistic and Diverse Practice in Podiatry

#### 3.2.1 Prioritise the Interconnectedness of Foot Health and a Person's Overall Health and Wellbeing

Students identified common and widespread public misconceptions about the podiatry profession, the importance of foot health and the significant role podiatry plays in overall health and wellbeing. They recognised that these misconceptions and negative stereotypes about what podiatrists do contribute to a stigma that undermines the profession's value. This general misunderstanding and lack of awareness in the community can influence a student's decision to pursue a career in podiatry. Addressing these misconceptions and actively promoting the full scope and impact of podiatry practice could help reduce the stigma and potentially attract more students to the discipline:

The public do not understand the importance of foot health and the positive impact this profession has on people's lives. If we can change the stigma around podiatry, and what we actually do, this may attract more students

(participant #476, F, fourth‐year podiatry student)

There is a prevalent misconception that podiatry is limited to care of the foot and is primarily pathology‐focused (i.e., rather than being holistic). Although podiatry programs adopt biopsychosocial models and emphasise holistic, person‐focused care, these counter‐beliefs persist:

In my uneducated opinion it feels like a small field with not a lot of scope for change and/or movement. I don’t have an interest in feet and it doesn’t align with my desire to work in a field where the person is viewed holistically as opposed to a particular body part

(participant #695, F, fourth‐year occupational therapy student)

Our quantitative data show that non‐podiatry students seem to have a good understanding of podiatry and their areas of expertise. In relation to clinical settings that a podiatrist can work in, 67.6% to 82.3% of the non‐podiatry students nominated each of the work settings. Similarly, non‐podiatry students seemed to be aware of podiatry's involvement in different specialities, with 70.5% to 75.9% of students identifying each of the speciality areas (Table 2). However, they seem to not realise the holistic scope of podiatry on a person's health. This perception of podiatry was further supported by quantitative data, whereby non‐podiatry students who reported a motivator of ‘wanting to make a difference to people's health’ were *less likely* to consider studying podiatry (OR 0.56, 95% CI 0.33 to 0.97, *p* = 0.036). Non‐podiatry students were *more likely* to have considered studying podiatry as a career option if they wanted an ‘opportunity to care for people from different backgrounds and age groups’ (OR 1.74, 95% CI 1.26 to 2.44, *p* < 0.001). However, they were *less likely* if they had prior qualifications (OR 0.53, 95% CI 0.28 to 0.99, *p* = 0.049) or were in their second year of study (OR 0.35, 95% CI 0.18 to 0.67, *p* = 0.002) (Table 3). The podiatry students noted that the discipline remains largely obscure until one enrols in a podiatry program and believe that there is a need for a greater emphasis on the interconnectedness of foot health and a person's overall health and wellbeing:

I think it’s not just about attracting people to podiatry but attracting people to areas (e.g., aged care, hospitals). People think it’s mostly sports, cutting nails. I think there needs to be more emphasis as well on what podiatrists can do for falls prevention and for lower limb injuries/problems. Most ankle injuries etc. still get referred to physio[therapy]—a lot of people still don't consider podiatrists because of the stigma that we cut nails

(participant #114, F, fourth‐year podiatry student)

**TABLE 2:** Student perspectives on marketing the podiatry profession.

		Podiatry		Non‐podiatry	*p*‐value^a^
Survey questions and responses	*N*	Descriptive statistic	*N*	Descriptive statistic	
Q15/Q16. In your opinion, to what extent is your podiatry [health/sport] course a rewarding career choice? *Mean* (SD)^b^	252	81.9 (15.7)	482	88.2 (11.7)	< 0.001^a^
Q16/Q17. Right now, how likely are you to recommend your podiatry [health/sport] course as a career? *Median* (IQR)^c^	254	3 (3, 4)	482	3 (3, 4)	< 0.001^a^
Q22/Q25. Compared to other allied health courses, how well do you think podiatry courses are promoted? *Mean* (SD)^d^	250	33.1 (23.4)	462	25.9 (18.3)	< 0.001^a^
Q23/Q26. Compared to other allied health courses, how well do you think career opportunities in podiatry are promoted? *Mean* (SD)^d^	248	42.2 (26.1)	461	26.0 (20.1)	< 0.001^a^
Q25/Q28. To what extent do you think the following social media platforms would be appropriate and/or effective to source information about podiatry [health/sport] as a career and the courses available? *Median* (IQR)^e^					
Instagram	248	3 (2, 4)	461	3 (2, 4)	0.594
Facebook	248	3 (3, 4)	461	3 (3, 4)	0.680
Twitter	248	2 (1, 3)	461	2 (1, 3)	0.473
TikTok	248	3 (2, 4)	461	3 (2, 4)	0.349
Snapchat	248	2 (1, 2)	461	1 (1, 2)	0.178
LinkedIn	248	3 (2, 4)	461	3 (2, 4)	0.281
Q26/Q29. To what extent do you think the following advertising platforms would be effective for the promotion of podiatry [health/sport] as a career and the courses available? *Median* (IQR)^e^					
University websites	245	4 (3, 4)	455	4 (3, 4)	0.313
Association websites	244	3 (2, 4)	455	3 (3, 4)	0.719
Career exhibitions and roadshows	245	4 (3, 4)	455	4 (3, 4)	0.268
Social media	245	4 (3, 4)	455	3 (3, 4)	0.006^a^
Career talks in high schools	245	4 (3, 4)	454	4 (3, 4)	0.878
Multi‐media	245	3 (2.5, 4)	455	3 (2, 4)	0.007^a^
Q23. To the best of your knowledge, podiatrists can work within the following settings (choose all that apply), *n* (%)					
Acute/Sub‐acute hospitals	—	—	553	374 (67.6)	—
Community health centres			553	401 (72.5)	
Private practice			553	455 (82.3)	
Aged care facilities			553	400 (72.3)	
Research/Academia			553	378 (68.4)	
Teaching/Clinical supervision			553	372 (67.3)	
Q24. To the best of your knowledge, podiatrists are involved in the following speciality areas (choose all that apply), *n* (%)					
Sports medicine and rehabilitation			553	390 (70.5)	
Paediatrics			553	367 (66.4)	
High‐risk foot (e.g., wound care and amputation prevention)			553	415 (75.0)	
Podiatric surgery (e.g., minor surgical procedures of the skin and nails)			553	416 (75.2)	
Geriatrics (i.e., providing care to elderly people)			553	400 (72.3)	
General podiatry (e.g., nail and skin care)			553	420 (75.9)	

*Note:* Data are mean (SD) or median (IQR), unless otherwise specified. Text outlined in square brackets corresponds to questions or responses specific to the non‐podiatry student survey.

^a^ Significant between‐group difference (*p* ≤ 0.05) based on the independent samples *t*‐test or Mann–Whitney *U* test (depending on data type).

^b^ Questions were answered on a 100‐point scale (0 = ‘not rewarding’ to 100 = ‘very rewarding’).

^c^ Questions were answered on a 4‐point Likert scale (1 = ‘extremely unlikely’; 2 = ‘unlikely’; 3 = ‘likely’ and 4 = ‘extremely likely’).

^d^ Questions were answered on a 100‐point scale (0 = ‘not well promoted’ to 100 = ‘very well promoted’).

^e^ Questions were answered on a 4‐point Likert scale (1 = ‘not at all’; 2 = ‘to a small extent’; 3 = ‘to a moderate extent’ and 4 = ‘to a great extent’).

**TABLE 3:** Multiple logistic regression analyses exploring factors associated with non‐podiatry students considering podiatry as a career choice.

Variables		Univariate			Multivariate		
	*N*	OR	95% CI	*p‐*value*	OR	95% CI	*p*‐value*
Age, *years*	473	0.99	0.96 to 1.01	0.319	0.97	0.93 to 1.02	0.246
Sex, *female*	473	0.70	0.44 to 1.13	0.135	0.48	0.27 to 0.87	0.016
Prior educational qualifications	473	0.72	0.46 to 1.10	0.135	0.53	0.28 to 0.99	0.049*
Carer responsibilities	473	1.02	0.58 to 1.76	0.934	1.92	0.91 to 4.04	0.085
Primary role in year prior to commencing program of study	473						
Final year of high school		—	—	—	—	—	—
Studying another course		1.01	0.56 to 1.77	0.982	1.12	0.54 to 2.25	0.762
Working		0.93	0.56 to 1.52	0.766	1.71	0.79 to 3.72	0.177
Undertaking a ‘gap year’		1.22	0.50 to 2.77	0.647	1.57	0.58 to 3.99	0.355
Other		0.99	0.14 to 4.49	0.993	1.78	0.21 to 11.10	0.554
Program of study	473						
Physiotherapy		—	—	—	—	—	—
Sport and exercise science		1.15	0.45 to 2.72	0.755	1.05	0.33 to 3.16	0.932
Occupational therapy		0.87	0.51 to 1.48	0.606	1.18	0.51 to 2.70	0.695
Speech pathology		3.03	0.35 to 25.90	0.276	3.10	0.29 to 33.80	0.329
Orthoptics		0.55	0.15 to 1.54	0.297	0.15	0.01 to 4.11	0.183
Prosthetics and orthotics		2.77	1.12 to 6.82	0.025*	1.13	0.08 to 28.30	0.928
Science		1.18	0.48 to 2.70	0.697	3.99	0.92 to 17.80	0.066
Other		0.85	0.41 to 1.71	0.666	1.82	0.54 to 6.07	0.329
Year level	473						
First		—	—	—	—	—	—
Second		0.46	0.26 to 0.81	0.008*	0.35	0.18 to 0.67	0.002*
Third		0.70	0.41 to 1.20	0.200	0.79	0.40 to 1.52	0.478
Fourth		0.57	0.29 to 1.07	0.086	0.56	0.26 to 1.17	0.131
Interest in a health‐related career	473	1.24	0.83 to 1.91	0.321	1.54	0.91 to 2.69	0.121
Interest in a sport‐related career	473	1.02	0.87 to 1.20	0.789	0.97	0.77 to 1.22	0.790
Wanting to make a difference to peoples’ health	473	0.94	0.63 to 1.42	0.745	0.56	0.33 to 0.97	0.036*
Opportunity to care for people from different backgrounds and age groups	473	1.40	1.08 to 1.82	0.012*	1.74	1.26 to 2.44	< 0.001*
Inspired by a health professional	473	0.99	0.83 to 1.19	0.904	0.96	0.77 to 1.21	0.726
Encouraged by a peer	473	0.94	0.78 to 1.13	0.509	0.90	0.69 to 1.16	0.408
Encouraged by a family member	473	1.05	0.88 to 1.25	0.579	1.09	0.86 to 1.37	0.491
Earning potential	473	1.10	0.90 to 1.36	0.343	1.28	0.98 to 1.69	0.068
Could not get into another course	473	1.22	0.95 to 1.57	0.117	1.30	0.96 to 1.77	0.092
Availability of scholarships and financial assistance	473	1.03	0.80 to 1.33	0.793	0.95	0.70 to 1.29	0.765
Multiple career options post‐graduation	473	0.92	0.77 to 1.10	0.354	0.92	0.72 to 1.17	0.480
Flexible working hours	473	0.90	0.75 to 1.09	0.282	0.89	0.69 to 1.16	0.393

*Note:* Data are OR (95% CI).

* *p* ≤ 0.05.

Among the non‐podiatry students, there is also a perception that podiatry is less impactful for patients and communities compared to other health disciplines:

I can make more [of an] impact on an individual or a community by studying physiotherapy or medicine

(participant #585, M, first‐year physiotherapy student)

To address these misconceptions, students emphasised that the podiatry profession needs to invest more effectively in public health education and marketing. These efforts should aim to position foot health as intrinsic to a person's overall health and wellbeing and ensure the podiatry discipline is ultimately comparable with other allied health professions:

I think for promoting podiatry, our role in 'treating the whole person' needs to be promoted. We consider the whole patient, their biomechanics, systemic function, nutrition status, age for stage‐related changes, skin health, pharmacology. But you only learn this as a student—the wider population need to be educated (and future students!)

(participant #114, F, fourth‐year podiatry student)

#### 3.2.2 Focus on the Desirable Aspects of a Podiatry Career

Students expressed that despite the inherent nature of working with feet, which may be less appealing compared to other areas of healthcare, a focus on promoting the varied and appealing opportunities of podiatry (e.g., sports podiatry), the hands‐on aspects and the use of cutting‐edge technology could enhance interest in the profession. Learning from other successfully marketed professions, the inclusion of a ‘glamorous’ aspect could make the field more attractive to prospective students:

Maybe when promoting podiatry, focus on aspects of the career that are desirable (such as move the focus away from feet) and instead focus on the broad range of career opportunities, travelling with your degree, sports‐related opportunities, option to own your own business, etc. Sports‐related content is usually very popular, so potentially highlighting that aspect of podiatry may be helpful? You can't really change the fact that feet will always be less appealing to work with than, for example, a shoulder

(participant #431, F, second‐year physiotherapy student)

Needs to be marketed as a ‘glamorous’ career choice. Even though physiotherapy isn't inherently a ‘glamorous’ profession, it is marketed in a way that attracts young adults of working with their favourite sports stars or running your own successful business. The paradigm of podiatry needs to be changed in the way that physiotherapy has

(participant #391, M, third‐year physiotherapy student)

Students highlighted that a career in podiatry can offer a range of desirable aspects that may appeal to individuals seeking both professional fulfilment and financial independence. The quantitative and qualitative data showed that one of the most notable attractions is the opportunity to own and operate a private practice. Podiatrists who choose this path can benefit from the autonomy of managing their own business, making decisions about services, client care and business operations:

Advertise the course to prospective students, advertise the earning and independent business potential

(participant #255, F, second‐year sport and exercise science student)

#### 3.2.3 Highlight Podiatry Success Stories and Clinical Excellence

Students noted that podiatry currently lacks a strong presence on social media compared to other health disciplines, which may be contributing to a lower level of awareness of the profession:

If you look on YouTube/TikTok, med[ical] students are constantly demonstrating the cool stuff they see/do

(participant #190, F, third‐year podiatry student)

A discrepancy in the visibility of different professions on social media platforms was identified, for example, a search for ‘physiotherapy’ yields significantly more content than one for ‘podiatry’:

When you search ‘physio[therapy]’ vs. ‘podiatry’ on these platforms there are significantly less posts. ‘[name omitted] Podiatry’ on TikTok is a great example ‐ more accounts like this are needed. They have gained a large following in a short amount of time ‐ people are clearly intrigued!

(participant #476, F, fourth‐year podiatry student)

The quantitative data showed that social media platforms Instagram and Facebook emerged as the most popular among podiatry students (73.4% for Instagram and 59.7% for Facebook) and non‐podiatry students (65.8% for Instagram and 59.7% for Facebook) (Table 1). Facebook and Instagram were also the top‐rated (i.e., moderate to great extent) social media platforms for sourcing information about health courses and careers. Compared to the non‐podiatry students, podiatry students were *more likely* to think that multimedia (OR 1.38, 95% CI 1.11 to 1.72, *p* = 0.005) and social media (OR 1.28, 95% CI 1.02 to 1.61, *p* = 0.036) were effective marketing platforms for the promotion of podiatry courses and careers (Table 4). Feedback from students also highlighted the potential of Instagram in particular, in raising awareness about the work of podiatrists:

Lots of reels on Instagram about ‘day in the life of’ and why podiatry is rewarding

(participant #544, F, first‐year occupational therapy student)

**TABLE 4:** Multiple logistic regression analyses exploring student perceptions on social media platforms and advertising platforms for the promotion of podiatry courses and careers.

Social media platforms		Univariate			Multivariate		
Variables	*N*	OR	95% CI	*p*‐value*	OR	95% CI	*p*‐value*
Age, *years*	830	1.00	0.98 to 1.02	0.877	0.98	0.96 to 1.00	0.093
Sex, *female*	831	0.61	0.44 to 0.83	0.002*	0.58	0.41 to 0.83	0.002*
Prior educational qualifications	830	1.30	0.97 to 1.74	0.077	1.50	1.03 to 2.19	0.035*
Instagram	709	1.02	0.87 to 1.20	0.800	0.94	0.76 to 1.16	0.557
Facebook	709	1.01	0.84 to 1.21	0.947	1.00	0.80 to 1.24	0.975
Twitter	709	1.07	0.92 to 1.25	0.387	1.01	0.83 to 1.22	0.935
TikTok	709	1.06	0.93 to 1.22	0.369	1.10	0.92 to 1.33	0.295
Snapchat	709	1.11	0.94 to 1.30	0.224	1.08	0.89 to 1.32	0.432
LinkedIn	709	1.08	0.94 to 1.24	0.291	1.08	0.92 to 1.26	0.357

Advertising platforms							
Variables							
Age, *years*	830	1.00	0.98 to 1.02	0.877	0.98	0.95 to 1.00	0.054
Sex, *female*	831	0.61	0.44 to 0.83	0.002*	0.57	0.40 to 0.82	0.002*
Prior educational qualifications	830	1.30	0.97 to 1.74	0.077	1.45	0.99 to 2.13	0.058
University websites	700	0.88	0.70 to 1.11	0.281	0.99	0.73 to 1.36	0.964
Association websites	699	0.91	0.76 to 1.09	0.304	0.92	0.73 to 1.15	0.454
Career exhibitions and roadshows	700	0.86	0.69 to 1.06	0.160	0.84	0.64 to 1.12	0.227
Social media	700	1.29	1.06 to 1.58	0.011*	1.28	1.02 to 1.61	0.036*
Career talks in high schools	699	0.93	0.75 to 1.16	0.505	0.85	0.64 to 1.12	0.239
Multi‐media	700	1.25	1.05 to 1.50	0.014*	1.38	1.11 to 1.72	0.005*

*Note:* Data are OR (95% CI).

* *p* ≤ 0.05.

Students reflected that the relative absence of podiatry on social media platforms represents a missed opportunity to educate the community in general and engage prospective students, including high school students and people considering a career change:

…we are living in a day and age where if it isn't on social media people don't know about it. There needs to be engagement of social media of what a ‘typical day’ for a podiatrist entails

(participant #190, F, third‐year podiatry student)

### 3.3 Theme 3. Enable Early Exposure and Experience of Podiatry Practice

#### 3.3.1 Broaden the Understanding of Target Audiences for Student Recruitment

Many students acknowledged a lack of awareness about podiatry as a career option, which may be preventing prospective students from considering the field:

I honestly did not even know podiatry was a course that was offered. I assumed they were doctors that chose to specialise in that area. I think just getting the word out there to let people know the course exists as an option would be a great start!

(participant #155, F, third‐year nursing student)

The quantitative data showed that compared to the non‐podiatry students, podiatry students were *more likely* to first hear about the profession from a family member (OR 1.50, 95% CI 1.05 to 2.13, *p* = 0.025) or different health professional (OR 1.99, 95% CI 1.22 to 3.22, *p* = 0.005) and *less likely* to receive this information from school teachers (OR 0.46, 95% CI 0.22 to 0.90, *p* = 0.029), professional websites (OR 0.18, 95% CI 0.06 to 0.43, *p* < 0.001) or during work experience (OR 0.49, 95% CI 0.30 to 0.77, *p* = 0.002; Table 5).

**TABLE 5:** Multiple logistic regression analyses exploring factors associated with first hearing about podiatry.

Variables		Univariate			Multivariate		
	*N*	OR	95% CI	*p*‐value*	OR	95% CI	*p‐*value*
Age, *years*	830	1.00	0.98 to 1.02	0.877	0.99	0.97 to 1.01	0.232
Sex, *female*	831	0.61	0.44 to 0.83	0.002*	0.64	0.46 to 0.88	0.007*
Prior educational qualifications	830	1.30	0.97 to 1.74	0.077	1.36	0.94 to 1.95	0.098
Work experience	831	0.53	0.33 to 0.81	0.005*	0.49	0.30 to 0.77	0.002*
Career counsellor	831	0.51	0.28 to 0.86	0.017*	0.59	0.32 to 1.05	0.080
School teacher	831	0.44	0.22 to 0.82	0.014*	0.46	0.22 to 0.90	0.029*
Family member	831	1.41	1.03 to 1.93	0.033*	1.50	1.05 to 2.13	0.025*
Friend	831	0.98	0.66 to 1.44	0.921	1.03	0.68 to 1.55	0.899
Healthcare professional^a^	831	1.05	0.77 to 1.44	0.741	1.09	0.77 to 1.53	0.630
Different health professional^b^	831	1.75	1.10 to 2.75	0.017*	1.99	1.22 to 3.22	0.005*
Social media	831	0.94	0.51 to 1.65	0.830	1.24	0.65 to 2.32	0.501
University open day	831	1.14	0.73 to 1.75	0.565	1.41	0.87 to 2.28	0.155
Career exhibitions and roadshows	831	0.80	0.40 to 1.51	0.505	1.08	0.51 to 2.18	0.842
Association website(s)	831	0.18	0.06 to 0.43	< 0.001*	0.18	0.06 to 0.43	< 0.001*
Other	831	0.93	0.59 to 1.43	0.745	0.91	0.55 to 1.50	0.725

*Note:* Data are OR (95% CI).

^a^ Health professional working in profession.

^b^ Health professional not working in profession.

* *p* ≤ 0.05.

Students emphasised the importance of promoting podiatry programs to high school students, suggesting that university events, such as open days, could play an important role in raising awareness and enhancing understanding of the profession. Course promotion to high school students requires a carefully considered narrative, which engages young people. Students believed that highlighting the podiatry scope of practice, employment opportunities and career pathways are relevant to engage a high school student who has limited or no experience of allied health:

… advertising should target younger audiences and school students as they aren't exposed to the profession as a patient often (unlike physiotherapy for example) about the attractive prospects of the profession/study and the employment opportunities possible

(participant #183, M, second‐year podiatry student)

Talk about career prospects and employment rates. That was what solidified my decision to pursue podiatry. Also, mention how broad the podiatry field is (e.g., paediatrics, high risk podiatry, aged care, education etc)

(participant #129, F, first‐year podiatry student)

Students also highlighted the need to provide information about the subjects and the different facets of podiatry that university programs include. This would provide high school students with an improved understanding of the scope of podiatry:

I think this is an opportunity for individual courses to have ‘Open Days’ to discuss in full the details of the course and give year 12 students a better understanding of what they will be studying

(workshop #1, first‐year podiatry student)

Students also emphasised the importance of health promotion to younger people to increase awareness and understanding of the podiatry profession. This approach not only familiarises young people with the role of a podiatrist but also helps to instil an understanding of the importance of foot health from an early age:

Doing health promotion in schools, even primary schools, like dentists and oral therapists do, so people grow up knowing what a podiatrist is

(participant #613, F, third‐year podiatry student)

Students emphasised that when marketing podiatry, it should not be assumed that the profession is known to everyone. One quote from an international student highlighted that the podiatry profession does not exist in some countries and therefore, knowledge of the profession may be lacking:

It would be good if there were more ways to introduce podiatry to the community or students, especially international students, about what podiatry is, what we do, advantages they can get etc.

(participant #339, M, third‐year podiatry student)

Students highlighted that targeting students in related fields could significantly enhance awareness and interest in podiatry. These students already on a healthcare or science career path may be open to exploring podiatry as an alternative or complementary discipline, especially if they are exposed to its unique scope and diverse opportunities:

Have opportunities for prospective students and students considering transferring courses to podiatry to attend a class or placement before they apply so they can decide if podiatry is the right career path for them

(participant #248, M, second‐year occupational therapy student)

When considering strategies to attract students to the field of podiatry, it is important to recognise the significant portion of mature‐aged students who choose this profession as a career change. Our data reveal that more than half of the students (59.9%) enrolled in podiatry programs did not come directly from high school, reflecting a strong interest among individuals seeking new professional pathways later in life. A similar trend was observed among non‐podiatry students, with 60.7% not enroling in their respective health‐related programs directly from high school. This highlights the need for enhanced marketing efforts, particularly through social media and other platforms, to better engage younger generations and raise awareness of podiatry as a viable career option. Over a third of podiatry students (40.3%) had concerns about studying podiatry upon enrolment and 34.2% had considered leaving the course. This response was lower among the non‐podiatry students, where only 26.6% had considered leaving their respective health‐related courses. Health or stress (17.3% and 14.3%), study/life balance (15.5% and 12.7%) and difficulties relating to workload (12.6% and 9.9%) were the most common reasons cited by both the podiatry and non‐podiatry students, respectively (Table 1). Older students were identified as having more complex circumstances to manage (e.g., family and caring commitments, existing careers and financial obligations). These factors presented barriers and made the decision to return to study more challenging for some students:

Older age, already full‐time working, wanting to buy property, unsure if to complete another 4‐year undergraduate or 2‐year Masters…

(participant #114, F, fourth‐year podiatry student)

#### 3.3.2 Enhance Awareness Through Direct Engagement With Prospective Students

Students believed that information provided to prospective students should clearly outline the scope of podiatry practice, course requirements, commitment expectations and opportunities for work‐integrated learning. One student suggested that helping prospective students to visualise the wide range of career paths available in podiatry upon graduation would be beneficial:

Demonstrating that you could focus on specific passions such as community health, aged care facilities, local or high‐level sporting clubs, dermatology. This would assist in giving a larger perspective of what the students can visualise in themselves when they are to graduate

(workshop #1, first‐year podiatry student)

Using a 4‐point Likert scale (1 = ‘not at all’ to 4 = ‘to a great extent’), our quantitative data showed that the top‐rated advertising platforms for sourcing information about health programs and careers were university websites, followed by career talks in high schools, career exhibitions and roadshows and social media. Both podiatry and non‐podiatry students rated each of these platforms a median score of 4 (IQR, 3 to 4). These data suggest that these advertising platforms are highly valued by students seeking information about various health professions, including podiatry, which was also supported by the student voice:

Definitely career talks/career exhibitions in high school as students do not know much about podiatry at all and will hence not consider it as a career

(participant #383, F, first‐year physiotherapy student)

Students expressed a strong preference for university open day sessions, career exhibitions and roadshows providing prospective students with a comprehensive understanding of the various career options available within the healthcare sector including podiatry. This approach would allow students to engage directly with professionals from different disciplines, ask questions and gain a clearer insight into the roles and responsibilities of each field. By offering this inclusive perspective, students believed that these sessions could better inform their decision‐making process and help them make more informed choices about their career paths:

More uni[versity] open day type sessions with all allied health there to speak to what they do

(participant #647, F, first‐year prosthetics and orthotics student)

In addition to academic staff, podiatry students were perceived as a valuable resource for prospective students to hear from someone with lived experience:

More exposure for podiatry would be great, especially if we can get involved with students at high school directly prior to their final years of high school. We need to ensure that we talk about the diverse skill set which comes from podiatry and the opportunities available after graduation. As from my experiences during high school other students and even teachers did not know what a podiatrist was and if they did, they only thought they performed general skin and nail care, however, many students are interested in sports or working in hospitals so if we can promote those skills more it might help to change the stereotype surrounding podiatry.

(participant #123, F, fourth‐year podiatry student)

Regarding career talks in high school, invite students that are currently undertaking podiatry to inform those high school students about the course and career opportunities

(participant #126, F, first‐year orthoptics student)

Our quantitative data show that although the majority of students perceived their career choices as rewarding, the non‐podiatry students rated their career choices as more rewarding than the podiatry students and they were also more likely to recommend their career to peers. On average, using a 100‐point scale (0 = ‘not rewarding’ to 100 = ‘very rewarding’), podiatry students rated their perceptions of career choice as less rewarding than the non‐podiatry students (81.9 [SD, 15.7] vs. 88.2 [SD, 11.7]; mean difference 6.3, *p* < 0.001). Non‐podiatry students were significantly more likely to recommend their course as a career compared to the podiatry students (*p* < 0.001). When asked about the promotion of podiatry courses and career opportunities relative to other allied health courses, both podiatry and non‐podiatry students rated the promotion poorly. On average, using a 100‐point scale (0 = ‘not well promoted’ to 100 = ‘very well promoted’), podiatry students rated the promotion of podiatry courses at 33.1 out of 100 (SD, 23.4) and career opportunities at 42.2 out of 100 (SD, 26.1). In comparison, non‐podiatry students rated the promotion of podiatry courses at 25.9 out of 100 (SD, 18.3) and career opportunities at 26.0 out of 100 (SD, 20.1). These ratings were significantly lower than those of the podiatry students (mean difference of 7.2 for courses and 16.2 for career opportunities; *p* < 0.001 (Table 2).

#### 3.3.3 Engage Prospective Students Through Observation and Experience

Many students thought that the value of spending time observing or working with a podiatrist prior to enrolment should not be underestimated in relation to influencing a prospective student's understanding of podiatry and its scope of practice. One student reflected:

The only reason I chose [my course] was because it sounded interesting during the uni[versity] open day I went to. Since starting [my course], I have started a job as a receptionist at a podiatry clinic which had given me an in‐depth insight into the work force. If more information had been given about the podiatry workforce and I had been more exposed to it during year 12, I may have considered it as an option to study

(participant #214, F, second‐year prosthetics and orthotics student)

Students highlighted that the profession’s practical, ‘hands‐on’ approach and/or the use of advanced technology and equipment could serve as compelling selling points for those contemplating a career in podiatry:

Selling the clinical aspect of the programs…is particularly relevant for hands‐on learners

(workshop #1, first‐year podiatry student)

Perhaps promotion of the technology and equipment used by podiatry might spark interest in people—other than thinking the job is all hands on

(participant #512, F, first‐year physiotherapy student)

### 3.4 Theme 4. Improve Course Entry Pathways and Flexibility

#### 3.4.1 Promote Scholarships and Financial Support

Financial barriers were identified as a common challenge for many prospective students, particularly with the increasing costs of higher education:

Podiatry is not offered in my country which meant that I was forced to study in Australia, thus the financial burden is too high

(participant #376, F, second‐year podiatry student)

Students believed that expanding government‐funded scholarships, grants and loan options could make podiatry courses more affordable and accessible to students from different socioeconomic backgrounds. Subsidised education, similar to programs that support teachers and healthcare professionals in rural or underserved areas, could be a potential solution for attracting high‐quality students to the podiatry profession and help to address the workforce needs:

…government subsidise the course or government funded promotion similar to doctors agreeing to practice in rural areas or teachers agreeing to teach at public schools

(participant #255, F, second‐year occupational therapy student)

#### 3.4.2 Reduce Barriers of Entry Into Podiatry Programs

Students identified that reducing barriers to entry into podiatry programs is crucial for attracting a diverse range of students, particularly mature‐age students, international students and transfer students. Mature‐age students often face unique challenges, including balancing family, work and study commitments. Depending on feasibility, it would seem that a flexible course structure would serve as an enabler for interested students to enrol in podiatry:

…[podiatry program] is not suited to parents…

(participant #450, F, third‐year podiatry student)

Get universities to make vastly better student timetables and/or more flexible study programs… to be more thoughtful about their use of student time and you might get more sign‐ups and/or less drop‐out

(participant #100, non‐binary/non‐conforming, second‐year podiatry student)

Recognising prior education and qualifications was found to be an important step in easing the transition for students from other fields and those who have previously completed a related course of study. Providing pathways for credit transfer could shorten the time needed to complete the program, making it more attractive to a wider pool of applicants:

The uni[versity] did not recognise my previous occupation as I am a mature‐age student and it was quite difficult getting accepted

(participant #310, M, third‐year podiatry student)

Distance to travel for study was also identified as a limiting factor, particularly for students living in regional or remote areas:

When I first finished year 12, podiatry was my number one preference, and while I was accepted, the location of the uni[versity] made it difficult for me to go and study

(participant #50, F, fourth‐year podiatry student)

## 4 Discussion

This is the first study to gather insights and perspectives from current podiatry and non‐podiatry students on marketing strategies aimed at promoting the podiatry profession and the courses available. In doing so, approaches that enhance the profession's appeal to prospective students to increase enrolments into entry‐level podiatry programs were identified. The findings indicate that addressing misconceptions about the discipline, engaging key stakeholders and expanding target audiences via a range of marketing platforms could enhance the visibility and appeal of podiatry as a career. Reducing barriers to entry and offering flexible study options could further attract and retain more students. This discussion provides actionable insights for improving the promotion of podiatry, highlighting key messages, ideal promoters and most effective platforms to reach target audiences. Ultimately, these efforts may support the sustainability and growth of the podiatry profession through increased enrolments in podiatry education programs.

### 4.1 Addressing Misconceptions and Enhancing the Appeal of Podiatry

Misconceptions about podiatry, particularly among non‐podiatry students, emerged as a significant theme in this study and our previous work [4]. Many students viewed the profession as limited to foot care, such as toenail trimming, and were surprised to learn about its broader scope of practice, including roles in musculoskeletal care, paediatrics, falls prevention and reducing lower limb related complications of chronic disease, such as diabetes. Addressing these misconceptions is critical to enhancing the profession's appeal. Efforts should focus on highlighting the diverse career opportunities within podiatry, such as its contributions to preventative healthcare, mobility improvement and quality of life. In addition to broadening perceptions, it is important to address negative assumptions about podiatry, particularly regarding salary expectations and ethical practices in private practice. Providing students with a realistic understanding of the profession's challenges and rewards is essential. Transparent information, such as the comparability of podiatry salaries with other allied health professions [22] and the consistently high professional standards maintained by podiatrists, can help dispel these misconceptions. For example, data from the Australian Health Practitioner Regulation Agency annual report 2022/2023 [23] indicate that complaints against podiatrists have remained stable or decreased, reflecting strong professional conduct within the field [23]. Highlighting these strengths could enhance the overall perception of podiatry and present it as a dynamic and rewarding career choice.

Although the majority of podiatry students perceived their career choice to be rewarding (81.9/100), it was rated significantly less compared to the non‐podiatry students (88.2/100). Non‐podiatry students were also significantly more likely to recommend their chosen field to others. These findings align with the ongoing challenge of negative perceptions around podiatry, often seen as limited to foot care or being less dynamic than other allied health professions [4]. Podiatry's relatively low visibility within the broader health sector contributes to this limited understanding, highlighting the need to more clearly showcase the profession's diverse career opportunities and full breadth of scope.

### 4.2 Enhancing Recruitment Through Targeted Marketing

Current marketing strategies for podiatry courses may not be reaching their full potential. Both podiatry and non‐podiatry students in this study perceived the promotion of podiatry courses and career opportunities as poor, with non‐podiatry students rating these promotional efforts significantly lower. This finding highlights the need for targeted strategies aimed at specific audiences, such as domestic high school students and international students, to improve awareness and appeal. Early engagement with these groups, particularly during critical career decision‐making periods, could positively influence perceptions of podiatry. For example, integrating foot health education into school curricula and conducting interactive school visits may help to demystify the profession and emphasise its vital role in overall health and wellbeing. These activities could foster a broader understanding of podiatry's scope and inspire prospective students to consider it as a dynamic and rewarding career choice.

Mature‐age students and course transfer students represent additional opportunities for targeted recruitment. These groups often bring valuable life experience and clear career goals, which can enrich the profession. For these students, recruitment strategies should be tailored to emphasise how their existing skills and knowledge could be applied in podiatry, in addition to highlighting career and study flexibility, opportunities for professional growth and the ability to make a meaningful impact in patient care. By broadening the potential candidate pool and addressing the unique needs of these groups, marketing efforts could present podiatry as an inclusive and rewarding career option for individuals from a wide range of personal and professional contexts.

### 4.3 Marketing Strategies to Increase Awareness of Podiatry

Historically, podiatry courses have been marketed through traditional methods such as university open days, career exhibitions and roadshows. These events typically feature a broad range of courses, leaving prospective students to ‘discover’ podiatry on their own. Our previous research [4] and studies from the United Kingdom [1, 2] identified a general lack of awareness among allied health students regarding the scope of practice, career opportunities, job prospects and earning potential in podiatry. This could explain our findings that podiatry is not a first‐choice career option for many, with 35.3% of podiatry students advising that it was not their first choice when applying to study, compared to just 11.5% of non‐podiatry students.

Although social media platforms, such as Instagram and Facebook, are commonly used by both podiatry and non‐podiatry students, more formal avenues, such as university websites, high school career talks and career exhibitions and roadshows, were rated as being more effective for promoting careers and courses. This suggests that although social media is widely used, this brief format may not always provide the depth of information needed for informed career decisions. However, platforms such as Instagram, Facebook and TikTok, present significant opportunities to reach prospective students and address misconceptions about the discipline. Content should aim to display podiatry as a dynamic and impactful career by highlighting its broader health impacts and diverse career pathways. For instance, students suggested videos featuring ‘a day in the life of a podiatrist’ or testimonials from podiatry practitioners working in areas, such as sports medicine, paediatrics or high‐risk foot care, may help to portray podiatry as a meaningful and rewarding career choice. By strategically leveraging social media alongside more formal promotional efforts, stakeholders could create a balanced approach to attract a wide range of prospective students.

### 4.4 Enhancing the Appeal of Podiatry Through Advocacy

Our study highlights the under‐explored potential for podiatry practitioners and podiatry students to serve as ambassadors for the profession. Podiatry students often first encounter podiatry through personal connections with someone in the profession, suggesting that first‐hand advocacy could play a crucial role in raising awareness and promoting the profession. Encouraging podiatrists and podiatry students to actively participate in career events or produce engaging social media content could enhance the profession's visibility and appeal. By showcasing the dynamic and impactful aspects of podiatry in their daily interactions, practitioners could inspire prospective students to view it as a meaningful and rewarding career choice. This approach reinforces the importance of a collective effort across the profession to promote its full potential.

Our survey results further revealed that podiatry students valued career prospects, such as owning a business, hospital work and job security, more highly than non‐podiatry students. These preferences highlight the importance of marketing podiatry's unique career pathways and opportunities for professional growth. Students emphasised that showcasing podiatry's flexibility and diverse healthcare settings could enhance its appeal. Drawing inspiration from professions, such as physiotherapy, which appear to have successfully marketed roles, such as working with athletes or managing private practices, podiatry could similarly position itself as a dynamic and entrepreneurial field. Presenting these aspects prominently may help attract a wider range of prospective students.

To modernise the image of podiatry and to shift the narrative away from a focus on feet, marketing strategies should emphasise its hands‐on nature, advanced technology and cutting‐edge techniques. Promoting roles, such as sports‐related care, opportunities for international practice and diverse work environments, could help breakdown negative stereotypes and broaden the profession's appeal. Highlighting the strong employment prospects, earning potential and pathways to business ownership further demonstrates the tangible benefits of a career in podiatry. The podiatry brand requires a makeover to showcase contemporary practice, dispel negative stereotypes and misconceptions and position it as a rewarding and forward‐thinking career choice to prospective students.

First‐hand exposure to the profession is another critical component in shaping prospective students' understanding of podiatry. Observing or working alongside a podiatrist could provide invaluable insights into the clinical and interpersonal aspects of the field and potentially influence student career choices. Universities and podiatry practitioners could collaborate to create structured opportunities for shadowing, clinical sessions or inter‐professional education activities. These experiences may help prospective students appreciate the breadth of podiatry's scope of practice, while empowering them to share these insights with peers and family, amplifying advocacy efforts.

### 4.5 Improving the Accessibility of Podiatry Programs

Improving the accessibility and attractiveness of podiatry programs is essential for addressing workforce shortages and expanding the pool of qualified practitioners. Financial challenges were highlighted as a significant barrier for many students, emphasising the need for expanded government‐funded scholarships, grants and loan options. A subsidy model, similar to those used for teachers and healthcare workers in rural areas, could be adapted for podiatry to attract high‐quality students while addressing workforce needs. Further, offering mentorship programs as part of financial support packages could help improve retention and provide additional career development for graduates. Podiatry apprenticeships, shown to be promising within the United Kingdom [3], could be explored in Australia and New Zealand for enhancing student recruitment and retention.

Reducing barriers to entry into podiatry programs were identified as an important strategy, particularly for mature‐age students, international students and those seeking to transfer into podiatry from other fields. International students require clear guidelines and support services to ensure qualification equivalency is clear, as uncertainty could dissuade them from pursuing podiatry in Australia or New Zealand. In addition, scholarships targeting international students could help alleviate the financial burden. Transfer students and those with prior qualifications also highlighted the need for improved recognition of prior study. Credit transfer pathways were identified as a factor in reducing the length and cost of podiatry programs, ultimately making the discipline more appealing.

Traditional university timetables and course structures do not always align with the needs of today's students. Greater flexibility, such as offering evening or weekend classes, part‐time or remote learning options (as appropriate) and intensive blocks for on‐campus practical skill training may help accommodate diverse lifestyles. Increased use of asynchronous and synchronous learning technologies, such as recorded lectures and skill demonstration videos, is also encouraged. Micro‐credentialing, short courses and undergraduate certificate programs could provide modular and accessible pathways into the profession. Revising curricula to align with current practice requirements, establishing satellite campuses and partnering with local healthcare facilities for work‐integrated learning placements could further reduce the impact of geographic distance and improve accessibility to podiatry study.

### 4.6 Limitations and Strengths

These findings should be viewed considering the study's limitations, as detailed within our previous publication [4], including: (i) some closed‐ended survey questions may have limited deeper responses, though free‐text options and qualitative workshops allowed exploration of student experiences and perceptions further; (ii) incomplete survey responses were excluded using casewise deletion from regression models, and the survey, though piloted with 30 first‐year podiatry students, was not validated; (iii) participation may have been affected by the COVID‐19 pandemic and competing educational demands; (iv) respondent ethnicity, including Indigenous status, was not recorded to protect respondent anonymity, potentially limiting generalisability in Australia and New Zealand [4]; (v) marketing strategies were derived solely from the student perspective, without input from the broader stakeholders, which may limit the scope of the recommendations and (vi) the average age of the participants was 25 years, which may skew findings towards younger perspectives, though 60.4% of the sample were mature‐age students who did not enrol in their courses immediately after high school.

The findings should also be considered in light of the study's strengths, including: (i) it is the first to explore marketing strategies for promoting podiatry through the student lens; (ii) it used a large sample size, comprising 278 podiatry students and 553 non‐podiatry students (across 13 disciplines), representing approximately 32% of podiatry students across Australia and New Zealand; (iii) the integration of quantitative and qualitative data revealed key themes (such as enhancing podiatry’s visibility, advocating its diverse practice, enabling early exposure and improving entry pathways) whereas qualitative data provided additional insights, particularly on the role of social media, to add richness to the quantitative data; (iv) it provides actionable insights for enhancing marketing strategies and campaigns in podiatry, which could serve as a benchmark for future initiatives and (v) the findings are not only relevant to podiatry programs in Australia and New Zealand but may also have implications for podiatry programs internationally who are facing similar workforce challenges, such as those in the United Kingdom [1, 2, 3].

### 4.7 Implications of Study Findings

The findings of this study offer significant implications for promoting the podiatry profession and its education programs. By identifying effective marketing strategies, this research provides valuable guidance for efforts aimed at increasing student enrolments, a critical factor for sustaining and expanding the podiatry workforce. A practical approach for universities to adopt some of the proposed marketing strategies, as supported by our study's data, could involve a coordinated redesign of existing podiatry courses, shifting towards more flexible hybrid models. These models would enable students to engage in online study, maintain part‐time employment, attend campus for intensive study blocks and avoid the financial and social challenges of relocation. In addition, universities could collaborate with podiatry peak bodies, such as the Australian Podiatry Association and Podiatry New Zealand, to strengthen outreach and advocacy through the following actions:

- Produce a series of short video narratives featuring alumni and podiatry practitioners to promote podiatry as a vital allied health discipline, dispel common misconceptions and highlight specific areas of practice;
- Develop a targeted social media strategy to engage diverse student cohorts, including domestic high school students, mature‐age students, international students and course transfer students, to promote podiatry as an attractive, flexible and financially rewarding career pathway;
- Foster mutually beneficial relationships among current students, alumni and industry partners to co‐create innovative promotional initiatives and cultivate a shared commitment to advocacy for the profession.

This study provides a valuable resource for universities in Australia and New Zealand (and perhaps more broadly) in designing marketing campaigns to enhance the visibility of podiatry programs and to highlight the diverse career opportunities within the profession. Key stakeholders, such as the Australian Podiatry Association and Podiatry New Zealand, may use these findings to create campaigns that resonate with prospective students. The broader impact of this research extends to the long‐term growth and sustainability of the podiatry profession through advancing research, advocating at the government level, meeting the ever‐increasing demand for patient care and generating meaningful employment opportunities. By fostering these areas, the podiatry profession can continue to evolve, thrive and maintain its relevance in an ever‐changing landscape.

## 5 Conclusions

In conclusion, the findings highlight that although podiatry is seen as a rewarding career by those who pursue it, there is much work to be done in raising awareness and promoting the full scope of practice and career opportunities within the field. By targeting specific audiences, leveraging social media more effectively, enabling early exposure to podiatry practice and empowering podiatrists and podiatry students to act as ambassadors, the profession can be promoted more effectively and attract a more diverse range of students. Addressing the negative stereotypes and misconceptions about podiatry and emphasising its role in holistic healthcare is key to reshaping the public's perception of the discipline and improving student recruitment. Improving course entry pathways, offering greater flexibility for study and reducing barriers to entry will enhance student recruitment and retention. Ultimately, these efforts are vital for increasing student enrolments, reducing attrition and ensuring the continued growth and sustainability of the podiatry profession in Australia and New Zealand.

## Author Contributions

**Michelle R. Kaminski:** conceptualisation, formal analysis, funding acquisition, investigation, methodology, project administration, visualisation, writing – original draft, writing – review and editing. **Caroline Robinson:** conceptualisation, formal analysis, investigation, methodology, writing – original draft, writing – review and editing. **Glen A. Whittaker:** conceptualisation, formal analysis, investigation, methodology, writing – review and editing. **Malia Ho:** conceptualisation, formal analysis, investigation, methodology, writing – original draft, writing – review and editing. **Daniel R. Bonanno:** conceptualisation, formal analysis, investigation, methodology, project administration, writing – review and editing. **Shannon E. Munteanu:** conceptualisation, investigation, methodology, writing – review and editing. **Mollie Dollinger:** investigation, writing – review and editing. **Sia Kazantzis:** investigation, writing – review and editing. **Xia Li:** formal analysis, writing – review and editing. **Ryan S. Causby:** conceptualisation, investigation, writing – review and editing. **Mike Frecklington:** conceptualisation, investigation, writing – review and editing. **Steven Walmsley:** conceptualisation, investigation, writing – review and editing. **Vivienne Chuter:** conceptualisation, investigation, writing – review and editing. **Sarah L. Casey:** conceptualisation, investigation, writing – review and editing. **Matthew Cotchett:** conceptualisation, formal analysis, investigation, methodology, writing – original draft, writing – review and editing.

## Ethics Statement

This study was approved by the La Trobe University Human Research and Ethics Committee (HEC21057), and ethics approval was also obtained from the following institutions via mutual acceptance applications: Auckland University of Technology (21/161), Central Queensland University (22978), Charles Sturt University (H21077), Southern Cross University (2021/043), University of Newcastle (H‐2021‐0276), University of South Australia (203889), University of Western Australia (2021/ET000372) and Western Sydney University (H14404). All participants provided informed consent prior to data collection.

## Consent

The authors have nothing to report.

## Conflicts of Interest

The authors declare no conflicts of interest.

## Supporting information

Supporting Information S1

## Data Availability

All data generated or analysed during this study are included in this published article.
