# Supplementary material for: Student Perspectives on Marketing the Podiatry Profession and Course Promotion: A Mixed Methods Study
Source: J Foot Ankle Res. 2025 Jul 21;18(3):e70063. doi: 10.1002/jfa2.70063 (PMC12279468; doi:10.1002/jfa2.70063)
Supplement: Supplementary file 1 — Supporting Information S1 [file JFA2-18-e70063-s001.pdf]

Student perspectives on marketing the podiatry profession and course promotion: a mixed methods study

Michelle R Kaminski, Caroline Robinson, Glen A Whittaker, Malia Ho, Daniel R Bonanno, Shannon E Munteanu, Mollie Dollinger, Sia Kazantzis, Xia Li, Ryan S Causby, Mike Frecklington, Steven Walmsley, Vivienne Chuter, Sarah L Casey, Matthew Cotchett

---

Additional File 1. Good Reporting of A Mixed Methods Study (GRAMMS) checklist

GRAMMS checklist [1]

ITEM	PAGE(S)
1. Describe the justification for using a mixed methods approach to the research question	9
2. Describe the design in terms of purpose, priority and sequence of methods.	13
3. Describe each methods in terms of sampling, data collection and analysis.	9-13
4. Describe where integration has occurred, how it has occurred and who has participated in it.	13
5. Describe any limitation of one method associated with the presence of the other method.	N/A
6. Describe any insights gained from mixing or integrating methods.	39-40

1. O'Cathain A, Murphy E, Nicholl J. The quality of mixed methods studies in health services research. J Health Serv Res Policy. 2008;13(2):92-8.
